# Running session-conditioned human serum lowers prostate cancer cell spheroid formation

**DOI:** 10.1007/s00432-025-06350-3

**Published:** 2025-10-18

**Authors:** Giulia Baldelli, Alice Avancini, Diana Giannarelli, Lorenzo Budel, Veronica Gentilini, Anita Borsati, Linda Toniolo, Asja Conti, Michele Milella, Federico Schena, Giorgio Brandi, Sara Pilotto, Mauro De Santi, Cantor Tarperi

**Affiliations:** 1 Hygiene and Public Health Unit, Biomolecular Sciences Department, University of Urbino Carlo Bo, Urbino, PU Italy; 2 Section of Innovation Biomedicine-Oncology Area, Department of Engineering for Innovation Medicine (DIMI), University of Verona and University and Hospital Trust (AOUI) of Verona, P. le L.A. Scuro 10, 37134 Verona, Italy; 3 Department of Neurosciences, Biomedicine and Movement, University of Verona, Verona, Italy; 4 Facility of Epidemiology and Biostatistics, G-STeP, Fondazione Policlinico Universitario A. Gemelli, IRCCS, Rome, Italy; 5 Biomedical, Clinical and Experimental Sciences, Department of Medicine, University of Verona, Verona, Italy

**Keywords:** Translational research, Prostate cancer, Running sessions, 3D cell culture, Spheroid formation, Tertiary prevention

## Abstract

**Purpose:**

Physical activity is associated with a lower mortality and recurrence risk in cancer, yet the underlying mechanisms remain unclear. This study aimed to evaluate the effects of running sessions on the tumorigenic potential of prostate cancer cells using a 3D in vitro model.

**Methods:**

Fifteen healthy males completed two outdoor running sessions (5 km and 10 km), interspersed by 1 month of wash-out time. Blood samples were collected before (PRE), immediately after (POST), and 3 h after (POST-3 h) sessions. Human serum (HS) samples were used to stimulate LNCaP and PC3 cell lines in 3D in vitro culture technique. The spheroid formation ability was quantified after 21 days of incubation, using GelCount.

**Results:**

In both prostate cancer cell lines, a reduction in spheroid number was shown, by both running sessions and in all timepoints considered (LNCaP cells: 5 km: − 23.8%; 10 km: − 5.6% POST HS; 5 km: − 37.8%; 10 km: − 34.8% POST-3 h HS; PC3 cells: 5 km: − 14%; 10 km: − 15.9% POST HS; 5 km: − 14.2%; 10 km: − 13% POST-3 h HS). The spheroid volume was reduced by 42.6% (5 km) and 51.1% (10 km) with POST-3 h HS, in LNCaP cells; no significant reduction was observed in PC3 cells. No differences were found between the running sessions, while higher muscle mass, cardiorespiratory fitness and age were associated with greater reductions in spheroid number and volume, especially in LNCaP cells.

**Conclusion:**

Running sessions reduce prostate cancer cell spheroid formation, especially in participants with higher physical fitness. Shorter running distances showed comparable effects to longer ones, highlighting practical implications for real-world exercise prescriptions in oncology.

**Supplementary Information:**

The online version contains supplementary material available at 10.1007/s00432-025-06350-3.

## Introduction

Prostate cancer is one of the most diagnosed malignancies in men worldwide, accounting for one in every 13 cancers diagnosed globally, and 14% of all male cancers (Bray et al. 2024). Over the past 5 years, epidemiological data have reported significant disparities in incidence and mortality rates between countries, with an increasing trend observed in at least nine nations (Schafer et al. 2025). Nevertheless, in many countries in Europe, mortality rates are declining, likely due to improvements in early detection and advances in treatment strategies (James et al. 2024). According to data from EUROCARE-5, the 5-year relative survival rate for prostate cancer in Europe is 83% overall, and 90% for men diagnosed between the ages of 55 and 64 years (Trama et al. 2015). However, the risk of biochemical recurrence remains significant, with studies estimating a recurrence rate of 30–50% within 10–20 years after initial treatment (Liesenfeld et al. 2017).

Therefore, this landscape highlights the high prevalence of the disease and the urgent need for effective strategies to reduce the recurrence risk.

In the past two decades, there has been an exponential increase in evidence supporting the role of physical activity in cancer. In prostate cancer exercise has been demonstrated to improve cardiorespiratory fitness, strength, and body composition, as well as to mitigate several treatment-related adverse events, including fatigue, cardiovascular impairments, and peripheral neuropathy, overall enhancing the quality of life (Campbell et al. 2019). Cohort studies suggested an inverse association between physical activity levels and both mortality and recurrence risk (Krane et al. 2018). Furthermore, a pivotal randomized controlled trial in colon cancer confirmed the statistically significant and clinically meaningful benefits of exercise on disease recurrence and survival outcomes (Courneya et al. 2025). Data from meta-analyses confirmed an inverse correlation between amounts of post-diagnosis physical activity, prostate cancer-specific mortality, and longer cancer-free survival (Benke et al. 2018; Bergengren et al. 2023; Cuthbertson et al. 2020; Mctiernan et al. 2019).

These findings pave the way for new approaches focused on deciphering how physical activity may reduce the risk of cancer progression with the final aim to develop personalized programs for patients with prostate cancer. This includes exploring how exercise modulates cellular mechanisms, immunological parameters and epigenetic biomarkers. In response to these important questions, translational research in this field has recently drawn attention. In particular, cellular models based on in vitro approaches represent useful tools for evaluating how lifestyle changes or single exercise sessions can impact on cancer cell proliferation (Bettariga et al. 2024a, b; Metcalfe et al. 2021; Orange et al. 2020). Several studies have described that physical activity induces systemic and serological changes, capable of affecting in vitro cancer cells proliferation and viability. However, most existing investigations explored exercise in controlled conditions (e.g., on treadmill or cycle-ergometer at pre-defined intensities), which, although valuable for standardization, may not fully capture the complexity of real-world settings where environmental factors, self-paced effort, and competitive context can influence the physiological response. In addition, many in vitro models exploit a 2D cell growth assay, which is unable to accurately simulate the physiological tumor microenvironment. To overcome this issue and better mimic the biological mechanisms of cancer single-cell dormancy and re-growth in vitro, we have optimized a 3D cell growth assay (soft-agar) to evaluate the effects of exercise-conditioned sera on cancer cell behavior (Baldelli et al. 2021, 2024; De Santi et al. 2019). This approach may also help clarify the most active exercise modality, intensity and duration, which are known modulators of the serological response during exercise and in the post-exercise recovery period (Metcalfe et al. 2021).

Therefore, this study aims to investigate the effect of the most common type of aerobic activity, i.e., running, performed in two sessions, 5 km (km) versus 10 km, on the modulation of spheroid formation capacity of two prostate cancer cell lines (LNCaP and PC3), using a soft-agar 3D culture model. Furthermore, we aim to analyze participants and running-session characteristics associated with spheroid number and volume.

## Materials and methods

### Study design and participants

The study applied a prospective, non-randomized design to explore the impact of two running sessions on the spheroid formation capacity of prostate cancer cell lines. It was conducted as part of the “Run for Science” (R4S) research project, annually carried out by the University of Verona, to investigate the effect of running on several health-related parameters and in several conditions (Lippi and Schena 2017). Between April 2024 and June 2025, we recruited 15 healthy males who usually ran at least 10 km and had medical clearance for exercise. Participants were excluded if they presented significant cardiovascular, metabolic, or renal disease, hypertension, a history of stroke, or any other condition that could be exacerbated by exercise. Ethical approval for the study was obtained from the Ethics Committee of the University of Verona (protocol no. CARP #16/2024), and all participants signed the informed consent.

### Physical performance assessment and exercise sessions

Within two weeks prior to the first running session, participants were asked to attend the laboratory facilities at the Sport Science Section of the University of Verona, to assess their maximal oxygen consumption and their lactate thresholds (LT1 and LT2) though a treadmill ramp and step test, respectively (Faude et al. 2009; Seiler 2009). Body composition was estimated using a bioelectrical impedance scale (Tanita RD-545 HR); anthropometric parameters (body weight and height) and sociodemographic information were collected with a dedicated questionnaire.

The two running sessions were performed outdoors in the morning, in a real-world setting around the facilities of the Sport Science Section. Participants were instructed to run to the best of their ability considering the distances, starting individually in a staggered manner every 5–10 min. Heart rate monitors were worn during the sessions to quantify exercise intensity, according to the data obtained from the treadmill step test. The heart rate time spent in different intensity zones during the session was also tracked (Faude et al. 2009; Seiler 2009). The first running session was the 10 km distance, performed during the R4S event, whereas the 5 km session was conducted approximately one month later. During this washout period, participants were asked to maintain their usual training routine.

### Serum collection

For both sessions, blood samples were collected at three time-points: before running (PRE), immediately after the exercise (POST), and 3 h later (POST-3 h). During the three-hour interval between the post-run and the 3-h post sample, participants attended educational sessions focused on healthy lifestyle habits. Participants were instructed to avoid alcohol, caffeine, and moderate-to-vigorous-intensity exercise the day before the acute session. At baseline, fasting blood samples were collected, after which participants were provided with a standardized breakfast, composed of 30–60 g of highly digestible carbohydrates, a small portion of fats from nuts, 10 g of proteins. Approximately 10 mL blood was stored in 5 mL Vacutainer serum tubes at each time point. Samples, allowed to clot at room temperature for 30 min, then centrifuged at 1800 rcf for 10 min, aliquoted and stored at − 80 °C.

### Cell cultures

LNCaP and PC3 prostate cancer cell lines were purchased from the American Type Culture Collection (ATCC, Rockville, MD, USA). Cells were cultured in Dulbecco’s Modified Eagle Medium (DMEM) high glucose, supplemented with 10% v/v fetal bovine serum (FBS), 1 × MEM Non-essential Amino Acid Solution, 2 mmol/L L-glutamine, 0.1 mg/mL streptomycin, and 0.1 U/L penicillin. Cells were maintained in a 37 °C, 5% CO_2_ humidified incubator for a maximum of 15 passages. To better mimic the physiological conditions for glucose concentrations, during the experiments, the culture medium was replaced by red phenol-free DMEM supplemented with 80 mg/dL glucose. Moreover, the culture medium was FBS-free and supplemented with 5% v/v of each human serum (HS) sample.

### Soft-agar 3D cell culture assay

**Fig. 1:**
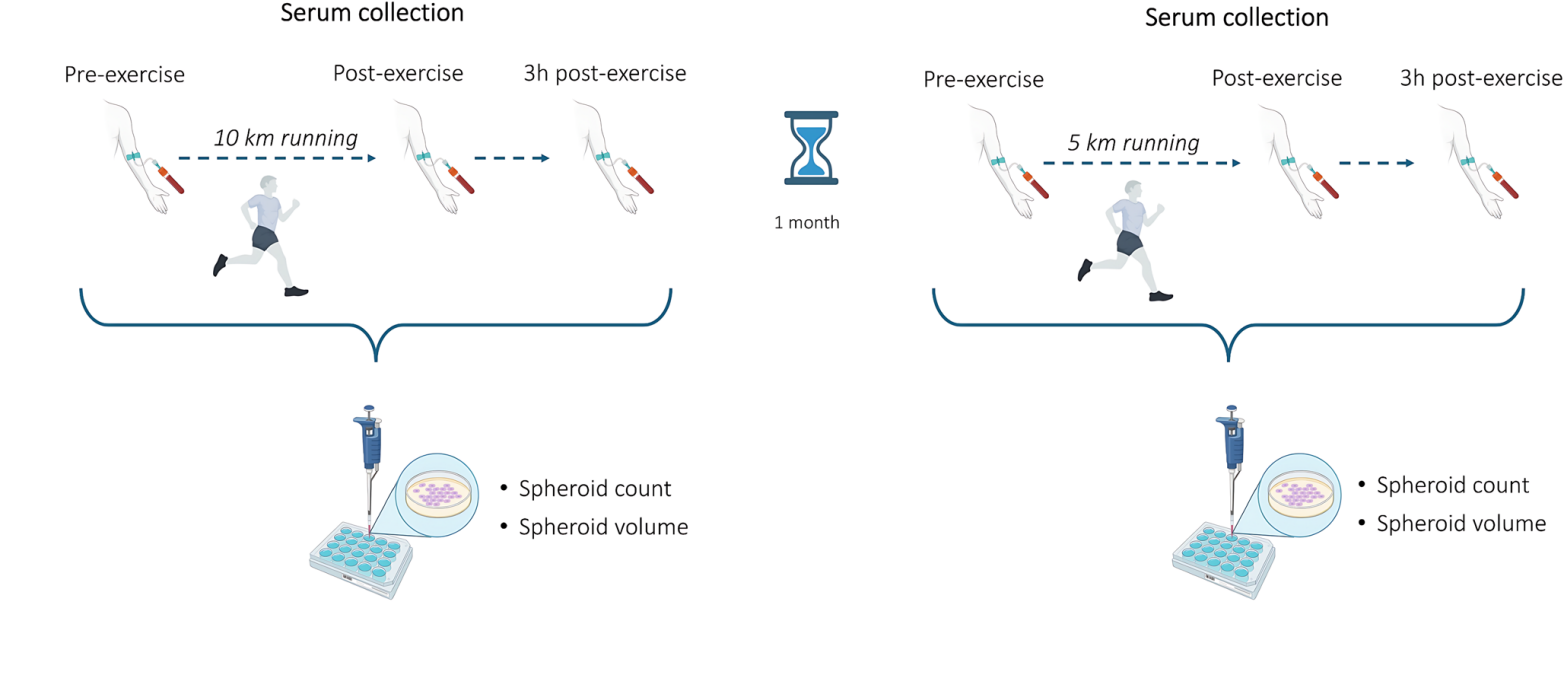
Assessment and procedures adopted in the study

The effect of exercise-conditioned HS on the tumorigenic potential of prostate cancer cells was evaluated in anchorage-independent culture conditions, to measure the 3D spheroid formation capacity. LNCaP and PC3 cells were grown in soft agar assay with 5% v/v HS samples collected at PRE, POST, and POST-3 h time-points, as previously reported (Baldelli et al. 2021, 2024; De Santi et al. 2019). Briefly, 1 × 10^3^ cells were cultured in 96-well plates in a 0.3% w/v agar layer, stimulated with each HS, and incubated for 21 days. Plates were then scanned through an automated high-throughput imaging and analysis system, GelCount (Oxford Optronix), quantifying spheroids according to their number and total volume, which is calculated as the sum of the product of optical density multiplied by area values (OD*µm^2^) of each identified spheroid. Specifically, the threshold of 50 µm for diameter was set for both cell lines; in contrast, two different minimal values was fixed for optical density of spheroids: 0.05 for LNCaP cells and 0.1 for PC3 cell line. The effects of HS are expressed as the means of a minimum of three independent experiments. Figure 1 shows the study design as described in the text.

### Statistical analysis

Data for all participants were summarized as mean and standard deviation (SD). Variations in LNCaP and PC3 prostate cells volume and counts were assessed with the repeated measurements ANOVA (Analysis Of Variance) and post-hoc comparisons were adjusted according to the Holm and Hochberg procedure. Multivariable regression analysis was used to adjust LNCaP and PC3 prostate cells volume and counts after exercise by baseline variables and running-session characteristics. Data analysis was performed using IBM-SPSS Statistics for Windows v.28.0 and R 4.4.1 (package *ggstatplot*).

## Results

### Participants' characteristics and sessions description

Fifteen participants joined the running sessions. Participants’ characteristics and running sessions parameters are shown in Table 1.

**Table 1:** Participants’ characteristics and running sessions parameters

Characteristics	Participants (n = 15)
*Participants characteristics*	
Age, mean (SD)	47.7 (16.4)
Body composition, mean (SD)	
Fat mass (%)	15.1 (5.4)
Muscle mass (kg)	58.6 (6.4)
Body mass index (kg/m^2^), mean (SD)	24.6 (2.3)
VO_2_max (ml/min/kg), mean (SD)	51.8 (7.7)
LT 1 (km/h), mean (SD)	10.0 (2.1)
LT 2 (km/h), mean (SD)	11.5 (2.6)
*Running session parameters*	
Intensity for the 5 km running session, mean (SD)	
Time spent under LT1 (%) for 5 km	0.7 (1.3)
Time spent between LT1 and LT2 (%) for 5 km	15.3 (33.1)
Time spent above LT2 (%) for 5 km	83.9 (33.2)
Intensity for the 10 km running session, mean (SD)	
Time spent under LT1 (%) for 10 km	5.4 (7.6)
Time spent between LT1 and LT2 (%) for 10 km	37.4 (37.8)
Time spent above LT2 (%) for 10 km	56.2 (42.2)
Time to complete the session, mean (SD)	
5 km (min.)	24.7 (5.0)
10 km (min.)	52.2 (10.6)

LT, lactate threshold; VO_2_max, maximal oxygen consumption

Overall, participants had a mean age of 47.7 ± 16.4 years, and the mean VO_2_max was 51.8 ± 7.7 ml/min/kg. They have completed the 5 and 10 km running sessions in approximately 24.7 and 52.2 min, respectively. Regarding intensity, more than half of the time was spent above the lactate threshold 2 (LT2) in both sessions.

### Exercise-conditioned serum and spheroid formation

**Fig. 2:**
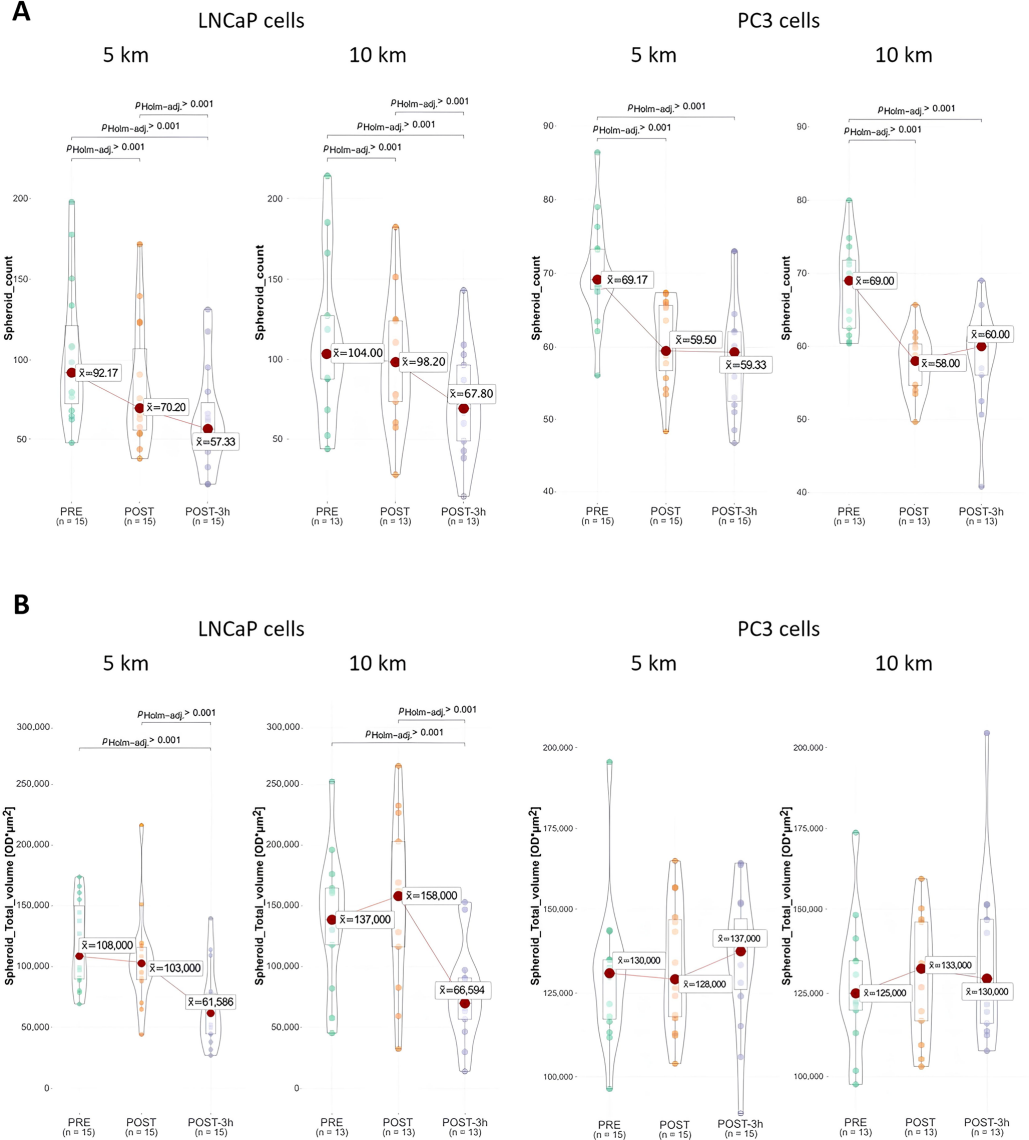
Spheroid formation according to running distance at different time-points. **A** shows spheroid count, by stimulating with PRE, POST, and POST-3 h human serum, according to running distance; **B** shows spheroid volume, by stimulating with PRE, POST, and POST-3 h human serum, according to running distance. PRE, before running session; POST, immediately post running session; POST-3 h, 3 h post running session

LNCaP and PC3 prostate cells were stimulated with HS collected at PRE, POST, and POST-3 h time-points of the 10 km and 5 km running sessions. Cells were grown in a 3D soft agar assay, and the spheroid formation parameters were assessed after 21 days of stimulation, using the GelCount instrument. Spheroid count and total volume results are shown in Fig. 2.

#### Post-exercise serum and spheroid count

Running session-conditioned HS samples reduce the tumorigenic capacity of prostate cancer cells (Fig. 2A). Focusing on the 5 km running session and LNCaP cells, the median spheroid number obtained by stimulating cells with PRE serum was 92.17, which was significantly reduced by 23.8% and 37.8% by culturing cells with POST and POST-3 h HS, respectively (*p* < 0.001). Similar results were obtained by stimulating LNCaP cells with 10 km-conditioned HS, in which the spheroid number reduction reached 34.8% with POST-3 h (*p* < 0.001). Analogously, a significant, although less marked, reduction in spheroid number was observed by stimulating PC3 cells with POST HS (5 km: − 14%; 10 km: − 15.9%) and POST-3 h HS (5 km: − 14.2%; 10 km: − 13%) (*p* < 0.001).

The spheroid counts and total volume obtained by stimulating prostate cancer cells with HS from each healthy donor are shown in Fig. S1 in the Supplementary Material.

Regarding LNCaP cells, after the 5 km-run, 12 out of 15 POST HS induced a reduction higher than 5% in spheroid number; after 10 km-distance, 9 out of 13 showed a similar decrease (*p* < 0.001). Intriguingly, this effect further improved when considering the POST-3 h sera. In this case, all the HS from the 5 km-run and 12 out of 13 of those collected in the 10 km-session induced a reduction of > 5% in spheroid number compared with PRE sera (*p* < 0.001). In PC3 cells, the effect on spheroid number is less evident but still significant, with a lowering of  > 5% in 12 out of 15 POST 5 km run HS, and in 12 out of 13 POST 10 km run HS (*p* < 0.001). The effect was analogous when PC3 cells were stimulated with POST-3 h HS, with 12 out of 15 and 9 out of 13 HS leading to spheroid count lowering  > 5%, in 5 km- and 10 km-running session, respectively (Fig. S1A) (*p* < 001, *p* < 0.01).

#### Post-exercise serum and spheroid volume

The variation of the spheroid total volume is reported in Fig. 2B. Considering the LNCaP cells, the trend was similar to that obtained for the spheroid count, particularly with POST-3 h serum. In the 5 km session, the median spheroid total volume with PRE serum was 1.08 × 10^5^ OD*µm^2^, which was reduced by 42.6% with POST-3 h HS (*p* < 0.001). Similarly, for the 10 km session, the median spheroid total volume with PRE serum was 1.37 × 10^5^ OD*µm^2^, which was decreased by 51.1% with POST-3 h HS (*p* < 0.001). Conversely, a slight but non-significant increase in total volume was observed in PC3 cells (*p* > 0.05).

**Fig. 3:**
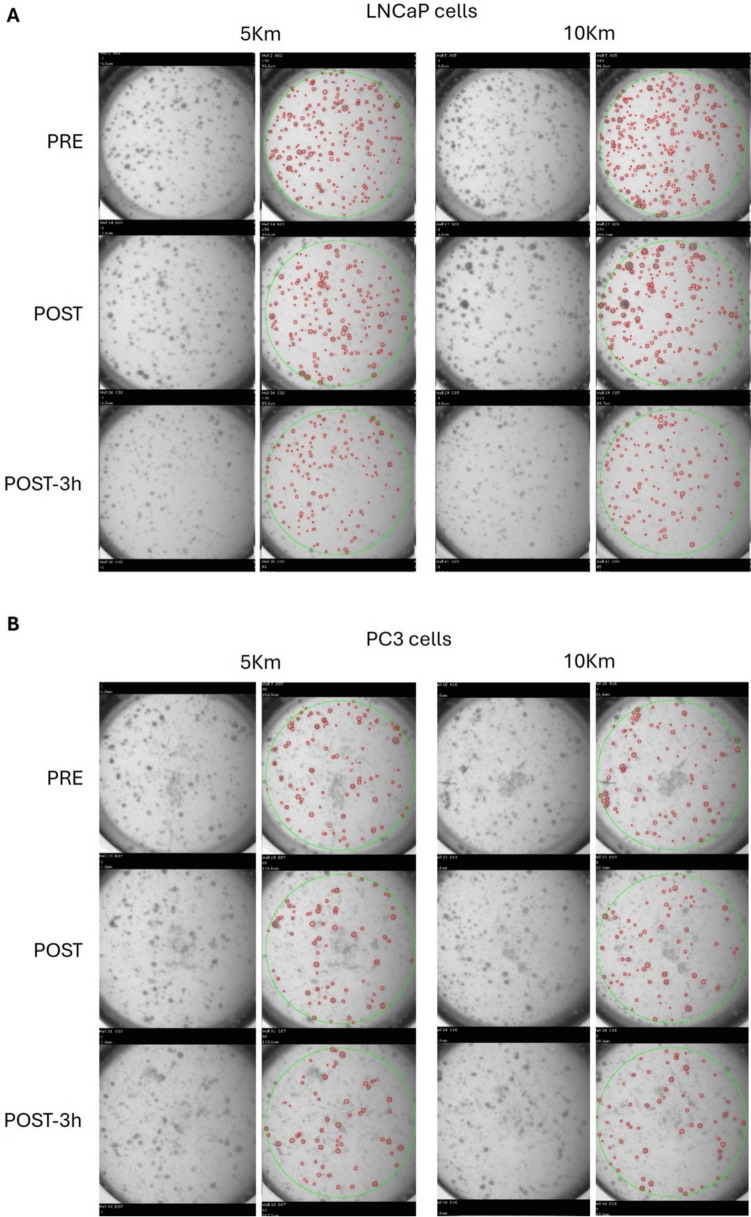
Representative scan image obtained through the automated high-throughput imaging and analysis system, GelCount (Oxford Optronix). The same image is shown as raw picture (left column) and as analyzed one (right column), in which red circles indicate the identified spheroids. PRE, before running session; POST, immediately post running session; POST-3 h, 3 h post running session

Interestingly, no differences were observed between the two running sessions in terms of spheroid count and total volume. Figure 3 shows a representative scan image; it points out the different spheroid morphology between LNCaP and PC3 cell lines. Particularly, LNCaP cells form well-defined, round-shaped and organized spheroids, whereas PC3 cells develop structures characterized by poor cell-to-cell contact, which in some cases result in more grape-like clusters.

The paired analysis of the spheroid total volume obtained by each HS showed a marked mean reduction in LNCaP cells, either stimulating cells with POST-3 h HS from 5 and 10 km-run (total volume reduction > 5% in 14/15 and 13/13 HS, respectively) (*p* < 0.001). The exploration of the possible modulation of the spheroid total volume of PC3 cells induced by the HS conditioned by the running distances did not reveal any statistical differences (Fig. S2B in the Supplementary Material).

### Determinants of spheroid count

A wide interindividual difference was noted in the number of spheroids from the stimulation of prostate cancer cells with the different PRE sera. The regression analysis (Table S1 in the Supplementary Material) revealed that among the participants’ characteristics, only BMI resulted in a significant effect on LNCaP cells spheroid count (β = − 13.974; *p* = 0.019). For PC3 cells, no predictors were found.

Table 2 shows the association between participants’ characteristics and LNCaP and PC3 cells count at POST and POST-3 h.

**Table 2:** Regression analysis analyzing participants and running-session characteristics associated with the count and volume of spheroid formation

Parameter	LNCaP cells count				PC3 cells count			
	POST		POST-3 h		POST		POST-3 h	
	β	Sig	β	Sig	β	Sig	β	Sig
Muscle mass (Kg)	− 1.456	.045	− 1.601	.024	− . 519	.000	.037	.871
BMI (kg/m^2^)	4.058	.096	.566	.817	1.145	.005	.126	.855
Age (yrs)	− . 814	.004	− 1.088	.000	− . 232	.000	− . 351	.002
VO2max (ml/min/kg)	− . 717	.264	− 1.934	.002	− . 478	.000	.194	.376
5 km running	− 16.512	.397	− 21.243	.302	− 5.243	.181	21.060	.005
10 km running	0ª		0ª		0ª		0ª	
Spheroid count at PRE	.788	.000	.496	.000	.676	.000	.947	.000
Time spent above LT2	.024	.759	− . 061	.436	.035	.032	.053	.048
Running time (s)	− . 006	.577	− . 012	.341	− . 004	.112	.015	.001

Parameter	LNCaP cells volume				PC3 cells volume			
	POST		POST-3 h		POST		POST-3 h	
	β	Sig	β	Sig	β	Sig	β	Sig
Muscle mass (Kg)	− 1105.3	.486	-2463.6	.004	− 74.3	.921	1516.6	.036
BMI (kg/m^2^)	4127.8	.403	1250.0	.647	− 515.2	.821	-857.5	.694
Age (yrs)	− 1471.2	.039	− 1743.8	.000	− 147.5	.640	139.8	.650
VO2max (ml/min/kg)	− 2246.3	.147	-3656.6	.000	− 82.5	.906	929.9	.172
5 km running	− 58,590.5	.173	− 34,191.9	.176	22,482.1	.266	38,328.2	.077
10 km running	0ª		0ª		0ª		0ª	
Spheroid volume at PRE	.705	.000	.310	.001	.612	000	.779	.000
Time spent above LT2	125.4	.491	− 40.9	.679	71.0	.416	26.4	.756
Running time (s)	− 15.4	.542	− 21.4	.166	15.9	.179	26.1	.051

^a^Set to zero because this parameter is redundant

PRE, before running session; POST, immediately post running session; POST-3 h, 3 h post running session

For LNCaP cells, POST and POST-3 h spheroid counts were inversely related with muscle mass (β = − 1.456; *p* = 0.045; and β = − 1.601; *p* = 0.024), age (β = − 0.814; *p* = 0.004; and β = − 1.088; *p* < 0.001) and positively associated with spheroid number obtained with PRE HS (β = 0.788; *p* < 0.001; and β = 0.496; *p* < 0.001). Additionally, VO_2_max was inversely associated with spheroid count at POST-3 h (β = − 1.934; *p* = 0.002). Focusing on PC3 cells, POST and POST-3 h spheroid counts were inversely related with age (β = − 0.232; *p* < 0.001; and β = − 0.351; *p* = 0.002) and directly associated with the PRE spheroid count (β = 0.676; *p* < 0.001; and β = 0.947; *p* < 0.001) and the time spent above the anaerobic threshold during running (β = 0.035; *p* < 0.001; and β = 0.053; *p* < = 0.048). Moreover, PC3 POST spheroid count was inversely related to muscle mass (β = − 0.519; *p* < 0.001), VO_2_max (β = − 0.478; *p* < 0.001), and positively associated with BMI (β = 1.145; *p* = 0.005). PC3 POST-3 h spheroid count resulted directly correlated with the 5 km running distance (β = 21.060; *p* = 0.005) and the time spent in the running sessions (β = 0.015; *p* = 0.001).

### Determinants of spheroid volume

Regression analysis evaluating the association between participants’ characteristics and PRE spheroid volume revealed no significant predictors (Table S2 in the Supplementary Material).

In contrast, the analysis revealed that POST and POST-3 h LNCaP spheroid volume were inversely related with age (β = − 1471.2; *p* = 0.039; and β = − 1743.8; *p* < 0.001) and positively associated with PRE spheroid volume (β = 0.705; *p* < 0.001; and β = 0.310; *p* = 0.001). Additionally, POST-3 h LNCaP spheroid volume was inversely correlated with muscle mass (β  = − 2463.6; *p* = 0.004) and VO_2_max (β = − 3656.6; *p* < 0.001). POST and POST-3 h PC3 spheroid volume were related with PRE spheroid volume (β = 0.612; *p* < 0.001; and β = 0.779; *p* < 0.001). POST-3 h PC3 spheroid volume was associated with muscle mass (β = 1516.6; *p* = 0.036).

## Discussion

Different guidelines for physical exercise in the oncological population have been published over the years (Avancini et al. 2025). Some, such as those of the American College of Sports Medicine, have aimed to develop more specific recommendations tailored to common side effects of cancer and treatment, such as anxiety, fatigue, depressive symptoms, health-related quality of life, and physical function (Campbell et al. 2019). However, most indications remain generic and pan-tumor. High-quality research is needed to support the development of optimized guidelines, considering the type, intensity, frequency, and duration of physical activity, according to the disease stage and features, the treatment type/phase, and clinical outcomes (Avancini et al. 2025).

In this context, the progression of in vitro cancer models has considerably improved the translational research landscape, providing valuable tools to start designing a personalized physical activity approach in exercise-oncology. Particularly, the application of 3D in vitro cancer cell models better mimics the oncogenic process and the mechanisms of prostate cancer recurrence, trying to bridge the gap between laboratory research and clinical application. To date, few studies have explored whether physical activity-conditioned HS can modulate prostate cell proliferation (Bettariga et al. 2024a, b). In addition, most of these investigations relied on 2D in vitro models, evaluating cell viability and proliferation, and employing only a single prostate cancer cell line. For instance, Rundqvist et al. demonstrated that HS collected 2 h after a single bout cycling (60 min at 50–65% of VO_2_max) induced a 31%-reduction in LNCaP proliferation and growth in 9 out of 10 healthy participants, compared with at rest serum (Rundqvist et al. 2013). In contrast, no impact on prostate cancer cell proliferation was observed in another study using the same protocol in twelve healthy donors (Hwang et al. 2020). As suggested by the authors, the results discrepancy could be primarily due to the different assays utilized to assess prostate cancer 2D cell growth, and different incubation conditions applied (times and culture media), emphasizing the limitations of bidimensional culture approaches in results consistency. To overcome this methodological issue, our research group was the first, to our knowledge, to apply the 3D soft agar in vitro assay to evaluate the effects of exercise-conditioned HS on the tumorigenic potential of prostate cancer cells. We previously reported that a high-intensity interval cycling, involving ten 90-s sprints at 90% of individual maximal power, led to a reduction in proliferation of LNCaP cells when stimulated with serum collected immediately, 4 h, and, interestingly, even 24 h post-exercise (Baldelli et al. 2021). However, the just-mentioned study evaluated only the effects of an acute bout of cycling, performed in standardized conditions of intensity and laboratory environment. To date, no evidence has explored the impact of other types of endurance activities that better mirror the real-world scenario.

In the current study, we aimed to address these gaps by collecting serum samples from healthy donors before, immediately after, and 3 h after two different running distances, 5 km and 10 km, to stimulate prostate cancer cell lines and evaluate their 3D growth in vitro. To better understand the role of running sessions in tertiary cancer prevention and to evaluate prostate cancer cell response to exercise-conditioned HS, two prostate cancer cell lines have been selected (LNCaP and PC3 cells). Particularly, LNCaP cells express androgen receptors (AR) and prostate-specific antigen (PSA), are androgen-dependent and similar to most of the prostate cancer encountered clinically; on the other hand, PC3 cells do not express AR and PSA, are androgen-independent, show highly aggressive behavior, possess higher migratory capability than LNCaP cells, and show features that are characteristic of prostatic small cell neuroendocrine carcinoma (Seim et al. 2017; Tai et al. 2011).

We found that, overall, 80% of HS collected immediately after the runs induced a reduction in prostate cancer spheroid number, and the effect was even more pronounced with HS obtained 3 h post-runs, with an average decrease in the spheroid number induced by 90% of sera. In detail, 5 km and 10 km running induced a reduction in LNCaP cell formation capacity by 23.8% and 5.6%, immediately post-exercise, and by 37.8% and 34.8% when stimulated with 3 h-post-exercise sera. Moreover, less pronounced, even if significant, was the mean effect on PC3 cells, where the reductions ranged from 13 to 15.9%. These findings are more favorable compared to those from our previous work and those reported by Rundqvist et al. (Rundqvist et al. 2013). Although speculative, one of the potential explanations includes the release of several exercise-induced factors, including catecholamine (e.g., epinephrine/norepinephrine), myokines and cytokines, androgens, and cortisol. As recently demonstrated by Safi and colleagues, even if low levels of systemic or intratumorally produced androgens can facilitate the control of prostate cancer growth and progression as a first-stage approach, a paradoxical mechanism of high dose androgens also exhibits considerable efficacy as a treatment modality in late-stage metastatic prostate cancer, facilitating the formation of AR dimers/oligomers to suppress c-MYC expression, and inhibiting proliferation (Safi et al. 2024). Indeed, the documented increase in testosterone and cortisol levels after a high-intensity exercise (De Luccia 2016) may contribute to the stronger reduction observed in the LNCaP cells, which are androgen-sensitive, compared to the PC3 cells, which are androgen-insensitive. Nevertheless, a recent in vitro study showed that testosterone nanoemulsion significantly reduced the growth and viability of both LNCaP and PC3 lines, although with a stronger impact on LNCaP, suggesting that the differences observed between the two cell lines are unlikely to be driven only by exercise-induced hormone modulation (Botelho and Queiroz 2024). However, the above-mentioned study used a different experimental method (2D metabolic activity analysis vs 3D in vitro model), which may limit the comparability of results. In any case, assessing possible changes in exercise-modulated hormones in HS samples collected in this study would be of interest for further projects.

Furthermore, another aspect that could be considered is the difference in p53 between LNCaP and PC3 cells. A known homozygous mutation in *TP53* was strongly identified in the PC3 cell line, with a further loss of p53 protein function, and the inhibition of the apoptosis pathway (Carroll et al. 1993; Seim et al. 2017). On the other hand, the involvement of increased level of p53 and apoptosis was identified as a possible mechanism implicated in the exercise-induced reduction of prostate cancer cells proliferation, with a 100% increase in p53 protein in LNCaP cells cultured with exercise-conditioned HS (Leung et al. 2004). Moreover, an additional concept that can arise from this aspect is the link between p53 and Insulin-like growth factor-I (IGF-1). IGF-1 contributes to the development of prostate cancer by stimulating cell proliferation and by inhibiting apoptosis. Furthermore, exercise is known to decrease the activation of IGF molecular axis, which can, in turn, enhance p53 action, initiating apoptosis (Leung et al. 2004). These aspects could further explain the fact that PC3 seems to be less sensitive to exercise-induced effects, exhibiting a lower reduction in cell spheroid formation capacity in response to exercise, in comparison to LNCaP cells.

An important finding is that no differences emerged between the two running distances. This may suggest that running 5 km is as effective as running 10 km. Nevertheless, analyzing more in depth the running session parameters shows that participants completed the 5 km running in an average of 24.7 min, and spent 83.9% of the time above the LT2 and 15.3% between LT1 and LT2. In the 10 km the mean running time was 52.2 min, and the percentage of time spent above LT 2 and between LT 1 and LT2, were 56.2% and 37.4%, respectively. From this perspective, the achieved training load was similar in the two running groups, potentially explaining the comparable results, in line with the literature highlighting how intensity and training load are essential in driving the exercise benefits (Bettariga et al. 2024a, b). From an exercise prescription point of view, these results are encouraging. While both the 5 km and 10 km runs were performed as all-out efforts, completing a 5 km distance is generally more attainable and requires a shorter time to achieve than a 10 km. This aspect may be particularly relevant for patients with cancer, who often experience physical and/or psychological limitations that could hinder their ability to engage in more demanding exercise sessions such as a 10 km run. In this sense, a 5 km distance may represent a more realistic and accessible target, while future studies should also explore interval-type training protocols, which more closely resemble typical training sessions than competitive races.

Another unanswered question in the literature is whether a person’s training status plays a role in influencing spheroid formation (Bettariga et al. 2024a, b). Our study may partially answer this question. The regression analysis showed that higher muscle mass and cardiorespiratory fitness were predictors of lower cell counts in both cell lines and lower spheroid volume in LNCaP cells, at different post-running time-points. Muscle mass and cardiorespiratory fitness are primarily shaped by chronic exercise. Regular running induces a series of physiological adaptations, promoting both skeletal muscle maintenance and an increase in cardiovascular function, enhancing oxygen delivery and metabolic efficiency (Hughes et al. 2018). This suggests that optimizing these physical fitness features through regular exercise may boost the positive effect of acute running sessions. Several biological mechanisms may sustain the protective effects of cardiorespiratory fitness and muscle mass, including enhanced metabolic regulation, reduced circulating insulin and IGF-1, which are key drivers of cancer cell proliferation (Tsiani et al. 2021), increased release of myokines, such as SPARC, OSM, IL‑6, and IL‑15, which have a direct antitumor effect by inhibiting cancer cells’ growth (Bettariga et al. 2025; Kim et al. 2022, 2023). Future studies are needed to further explore these underlying mechanisms.

In this project, we apply the translational approach to assess the effects on spheroid total volume. We found that in the LNCaP model, the spheroid volume was significantly reduced when stimulated with 3 h-post-exercise sera of 5 km or 10 km, whereas the effect on PC3 cells was less evident. This discrepancy may be related to the observed differences in spheroid growth shape and cell lines characteristics.

Another possible explanation is that PC3 cells appeared less sensitive to low doses of hormones (Botelho and Queiroz 2024); in this case, we can hypothesize that the sera in which exercise did not induce an extensive hormonal change, lead to a lower number of spheroids formed, but do not modulate their volume. Moreover, the loss of function of p53 in this cell line further could help us to comment and discuss the obtained results.

To our knowledge, this is the first study to assess the impact of exercise-conditioned sera on spheroid volume. These findings provide novel insights into the potential anti-tumorigenic impact of running. Spheroid volume is a recognized indicator of cell proliferation, viability, and aggressiveness, within a three-dimensional microenvironment that closely resembles the in vivo tumor architecture. Therefore, volume reduction may mirror a decrease in cancer cell growth (Friedrich et al. 2009; Vinci et al. 2012). From this perspective, these results reinforce the evidence that an acute bout of exercise, in this case running, may trigger systemic changes able to reduce cancer cell expansion and aggressiveness. Future studies are needed to validate these findings and elucidate the underlying mechanisms. On the other hand, these results emphasize the value of 3D in vitro models for exploring the complex interplay between tumor biology and systemic parameters (Baldelli et al. 2024).

Study limitations should be acknowledged. First, we included healthy and trained subjects, thus limiting the generalizability of our findings to patients affected by prostate cancer. Second, we did not randomize the running sessions, which may have introduced an order effect or learning effect. Third, the methodology approach showed a slightly different response in spheroid formation by the prostate cancer cell lines used, complicating the results comparison. Lastly, even if strongly significant, the results derived from a quite small sample size, with the need to consider a larger number of participants in further studies, to obtain more robust data. Nevertheless, evidence from this study may inform the planning of future experimental models involving larger cohorts, as well as patients with prostate cancer, to further refine the identification of exercise-modulated parameters associated with the prevention of disease recurrence. Moreover, the proposed model may be translatable to other tumor settings, offering a broader framework for investigating the impact of exercise on tumor biology. Ultimately, these insights could contribute to developing personalized exercise oncology strategies tailored to individual patient profiles and tumor characteristics.

## Conclusion

Overall, both 5 km and 10 km running sessions were effective in reducing the spheroid count in LNCaP and PC3 cells. Particularly, higher muscle mass and cardiorespiratory fitness were associated with greater reductions in spheroid number and volume, especially in LNCaP cells.

For the first time, we also observed a potential impact on spheroid volume. These findings offer novel insights and knowledge and support observational data on the beneficial effects of exercise in patients with prostate cancer.

## Supplementary Information

Below is the link to the electronic supplementary material.

Supplementary file1 (DOCX 279 KB)

Supplementary file2 (DOCX 19 KB)

## Data Availability

The datasets generated during and/or analysed during the current study are available from the corresponding author on reasonable request.
