# Supplementary material for: Running session-conditioned human serum lowers prostate cancer cell spheroid formation
Source: J Cancer Res Clin Oncol. 2025 Oct 18;151(12):297. doi: 10.1007/s00432-025-06350-3 (PMC12535562; doi:10.1007/s00432-025-06350-3)
Supplement: Supplementary file 1 — Supplementary file1 (DOCX 279 KB) [file 432_2025_6350_MOESM1_ESM.docx]

**Supplementary Figure**

**Title:** Running Session-Conditioned Human Serum Lowers Prostate Cancer Cell Spheroid Formation

**Authors:** Giulia Baldelli^1^*, Alice Avancini^2,3^*, Diana Giannarelli^4^, Lorenzo Budel^3^, Veronica Gentilini^1^, Anita Borsati^3,5^, Linda Toniolo^3^, Asja Conti^1^, Michele Milella^2^, Federico Schena^3^, Giorgio Brandi^1^, Sara Pilotto^2^, Mauro De Santi^1#^_,_ Cantor Tarperi^3#^

* Share the co-first authorship

# Share the co-last authorship

**Corresponding author:**

Prof. Sara Pilotto, MD., Ph.D.,

Section of Innovation Biomedicine - Oncology Area, Department of Engineering for Innovation Medicine (DIMI), University of Verona, Italy, P. le L.A. Scuro 10, 37134, Verona, Italy, ph. +39-0458128247, fax. +39-0458128140; e-mail: [sara.pilotto@univr.it](mailto:sara.pilotto@univr.it)

ORCID: <https://orcid.org/0000-0003-2229-4874>

**Journal:** Journal of Cancer Research and Clinical Oncology


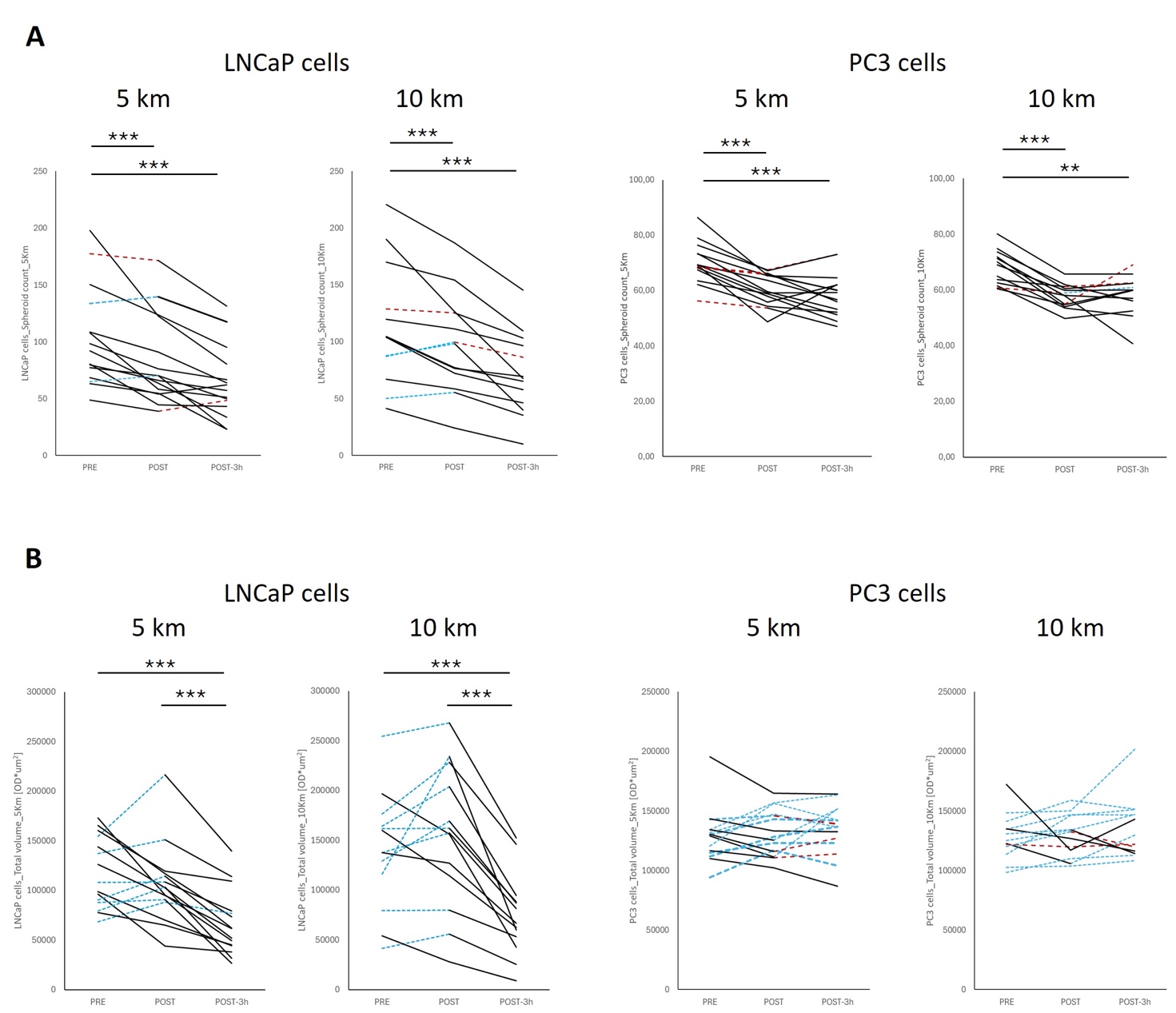


**Fig. S1** Spheroid count (A) and total volume (B) obtained by stimulating PC cells with huma serum at PRE, POST, and POST-3h from each healthy donor. Black lines indicate a reduction >5% in comparison with PRE serum; red lines indicate no modulation with respect to PRE serum samples; light-blue lines indicate an increase in comparison to PRE serum samples. Paired t-test; *** p< 0.001; ** p< 0.01. PRE, before running session; POST, immediately post running session; POST-3h, 3 hours post running session
