# Supplementary material for: Running session-conditioned human serum lowers prostate cancer cell spheroid formation
Source: J Cancer Res Clin Oncol. 2025 Oct 18;151(12):297. doi: 10.1007/s00432-025-06350-3 (PMC12535562; doi:10.1007/s00432-025-06350-3)
Supplement: Supplementary file 2 — Supplementary file2 (DOCX 19 KB) [file 432_2025_6350_MOESM2_ESM.docx]

**Supplementary Tables**

**Title:** Running Session-Conditioned Human Serum Lowers Prostate Cancer Cell Spheroid Formation

**Authors:** Giulia Baldelli^1^*, Alice Avancini^2,3^*, Diana Giannarelli^4^, Lorenzo Budel^3^, Veronica Gentilini^1^, Anita Borsati^3,5^, Linda Toniolo^3^, Asja Conti^1^, Michele Milella^2^, Federico Schena^3^, Giorgio Brandi^1^, Sara Pilotto^2^, Mauro De Santi^1#^_,_ Cantor Tarperi^3#^

* Share the co-first authorship

# Share the co-last authorship

**Corresponding author:**

Prof. Sara Pilotto, MD., Ph.D.,

Section of Innovation Biomedicine - Oncology Area, Department of Engineering for Innovation Medicine (DIMI), University of Verona, Italy, P. le L.A. Scuro 10, 37134, Verona, Italy, ph. +39-0458128247, fax. +39-0458128140; e-mail: [sara.pilotto@univr.it](mailto:sara.pilotto@univr.it)

ORCID: <https://orcid.org/0000-0003-2229-4874>

**Journal:** Journal of Cancer Research and Clinical Oncology

**Table S1**. Regression analyses using spheroid count obtained stimulating PC cells with human sera taken at rest (PRE) as dependent variables.

|  | **LNCaP cells** | | |  | | **PC3 cells** | |
| --- | --- | --- | --- | --- | --- | --- | --- |
|  | PRE |  |  | | PRE | |  |
| **Parameter** | B | *Sig.* |  | | B | | *Sig.* |
| **Muscle mass (Kg)** | 3.194 | *n.s.* |  | | -.018 | | *n.s.* |
| **BMI (kg/m²)** | -13.974 | *0.019* |  | | .087 | | *n.s.* |
| **Age (yrs)** | .474 | *n.s.* |  | | .298 | | *n.s.* |
| **VO2_max (ml/min/kg)** | -.682 | *n.s.* |  | | .376 | | *n.s.* |

PRE, before running session.

**Table S2**. Regression analyses using spheroid total volumes obtained stimulating PC cells with human sera taken at rest (PRE) as dependent variables.

|  | **LNCaP cells** | | |  | | **PC3 cells** | |
| --- | --- | --- | --- | --- | --- | --- | --- |
|  | PRE |  |  | | PRE | |  |
| **Parameter** | B | *Sig.* |  | | B | | *Sig.* |
| **Muscle mass (Kg)** | 1087.447 | *n.s.* |  | | -1072.618 | | *n.s.* |
| **BMI (kg/m²)** | -5476.690 | *n.s.* |  | | 2619.803 | | *n.s.* |
| **Age (yrs)** | -780.419 | *n.s.* |  | | -219.167 | | *n.s.* |
| **VO2_max (ml/min/kg)** | -1353.501 | *n.s.* |  | | 645.740 | | *n.s.* |

PRE, before running session.
